# Preclinical models of middle cerebral artery occlusion: new imaging approaches to a classic technique

**DOI:** 10.3389/fneur.2023.1170675

**Published:** 2023-06-20

**Authors:** Jennifer D. Sokolowski, Sauson Soldozy, Khadijeh A. Sharifi, Pedro Norat, Kathryn N. Kearns, Lei Liu, Ashley M. Williams, Kaan Yağmurlu, Panagiotis Mastorakos, G. Wilson Miller, M. Yashar S. Kalani, Min S. Park, Ryan T. Kellogg, Petr Tvrdik

**Affiliations:** ^1^Department of Neurological Surgery, University of Virginia, Charlottesville, VA, United States; ^2^Department of Neurological Surgery, Westchester Medical Center, Valhalla, NY, United States; ^3^Department of Neuroscience, University of Virginia, Charlottesville, VA, United States; ^4^Department of Neurological Surgery and Neuroscience, Northwestern University, Chicago, IL, United States; ^5^School of Medicine, Morsani College of Medicine, Tampa, FL, United States; ^6^Department of Neurological Surgery, University of Tennessee, Memphis, TN, United States; ^7^Department of Neurological Surgery, Thomas Jefferson University, Philadelphia, PA, United States; ^8^Department of Radiology and Medical Imaging, University of Virginia, Charlottesville, VA, United States; ^9^Department of Neurological Surgery, St. John's Neuroscience Institute, Tulsa, OK, United States

**Keywords:** ischemic stroke, middle cerebral artery occlusion, focal ischemia, rodent model, penumbra, core

## Abstract

Stroke remains a major burden on patients, families, and healthcare professionals, despite major advances in prevention, acute treatment, and rehabilitation. Preclinical basic research can help to better define mechanisms contributing to stroke pathology, and identify therapeutic interventions that can decrease ischemic injury and improve outcomes. Animal models play an essential role in this process, and mouse models are particularly well-suited due to their genetic accessibility and relatively low cost. Here, we review the focal cerebral ischemia models with an emphasis on the middle cerebral artery occlusion technique, a “gold standard” in surgical ischemic stroke models. Also, we highlight several histologic, genetic, and *in vivo* imaging approaches, including mouse stroke MRI techniques, that have the potential to enhance the rigor of preclinical stroke evaluation. Together, these efforts will pave the way for clinical interventions that can mitigate the negative impact of this devastating disease.

## Introduction

In the United States and worldwide, stroke is one of the leading causes of morbidity and mortality (1). Risk factors associated with stroke include age, hypertension, hyperlipidemia, cardiac disease, smoking, and diabetes (2–4). While many of these factors are modifiable, the risk and prevalence of stroke is expected to rise given the aging populations (2). In addition, there is a large cost burden associated with stroke, with direct medical costs estimated to be $17.9 billion, and up to $33.9 billion when factoring in indirect costs (1).

Strokes can be categorized as either ischemic or hemorrhagic, constituting 87 and 13% of strokes, respectively (1). Most ischemic strokes are a result of acute vessel occlusion from a thrombotic and/or embolic event, which causes transient or permanent hypoperfusion. Hypoperfusion can have varying effects; the magnitude of injury is determined by the degree to which the territory is reliant on perfusion from the occluded vessel, the duration of ischemia, and the metabolic demands of the tissue and its relative resistance to insult. First, there are regions with *benign oligemia*, in which perfusion is decreased but the functional and structural integrity of the brain are preserved. Second, there is a region of *penumbra* that is considered “at-risk” tissue, characterized by a loss of function without permanent structural damage. Therapeutic interventions aim to save these regions from devolving into infarct *core*. The ischemic *core* is the region where irreversible damage has occurred, and tissue is not salvageable.

Fundamental research is warranted to optimize management and explore new therapeutic interventions, and relevant animal models are mandatory to evaluate novel therapeutic strategies. Here, we review the middle cerebral artery occlusion (MCAo) techniques that are widely used in stroke research in rodents. We also highlight newly emerging approaches for *in vivo* imaging and staining that have the potential to provide more nuanced insights into the pathophysiology of stroke, as well as serve as indices for testing the effects of putative therapies for this devastating disease.

## Rodent models of focal ischemia

Clinically, the most common site of ischemia occurs within the middle cerebral artery (MCA) territory, therefore MCA occlusion (MCAo) is a popular technique for experimentally-induced injury (5). There are a number of different rodent models for MCAo, each with its own advantages, disadvantages and limitations (summarized in Table 1).

**Table 1:** Animal models of focal ischemia.

Models of focal ischemia	Technique	Advantages	Disadvantages	References
MCAo via filament	Filament inserted into vessel to cause occlusion. Approach is via an anterior cervical to expose the common carotid. Filament is advanced through an arteriotomy in the CCA to occlude the MCA origin.	High reproducibility. Can be permanent or transient. Does not require craniotomy.	The occlusion of the MCA is not visualized. Area of infarct can be variable depending on blood vessel anatomy.	(6, 7)
MCAo via cranial window	Coagulation or clipping of MCA branch. Approach is via a craniotomy over the MCA.	Successful MCAo can be visualized. Can be used for transient or permanent ischemia.	Technically challenging procedure. Invasive craniectomy	(8, 9)
Thrombin injection model	Intraluminal microinjection of thrombin. Approach is via a craniotomy over the MCA.	Responds well to tPA. Activates inflammatory cascades.	Requires craniectomy. Doppler laser measurement of blood flow might be needed to assess for successful stroke.	(10)
Photothrombosis model	Systemic injection of photosensitive dye such as Rose Bengal or erythrosine B combined with focal photoactivation to induce thrombosis.	High accuracy of ischemic area using stereotactic coordinates to target the radiating light.	Can cause early vasogenic edema that is uncharacteristic in human stroke. Invasive Craniectomy.	(11, 12)

### Proximal middle cerebral artery occlusion

**Figure 1:**
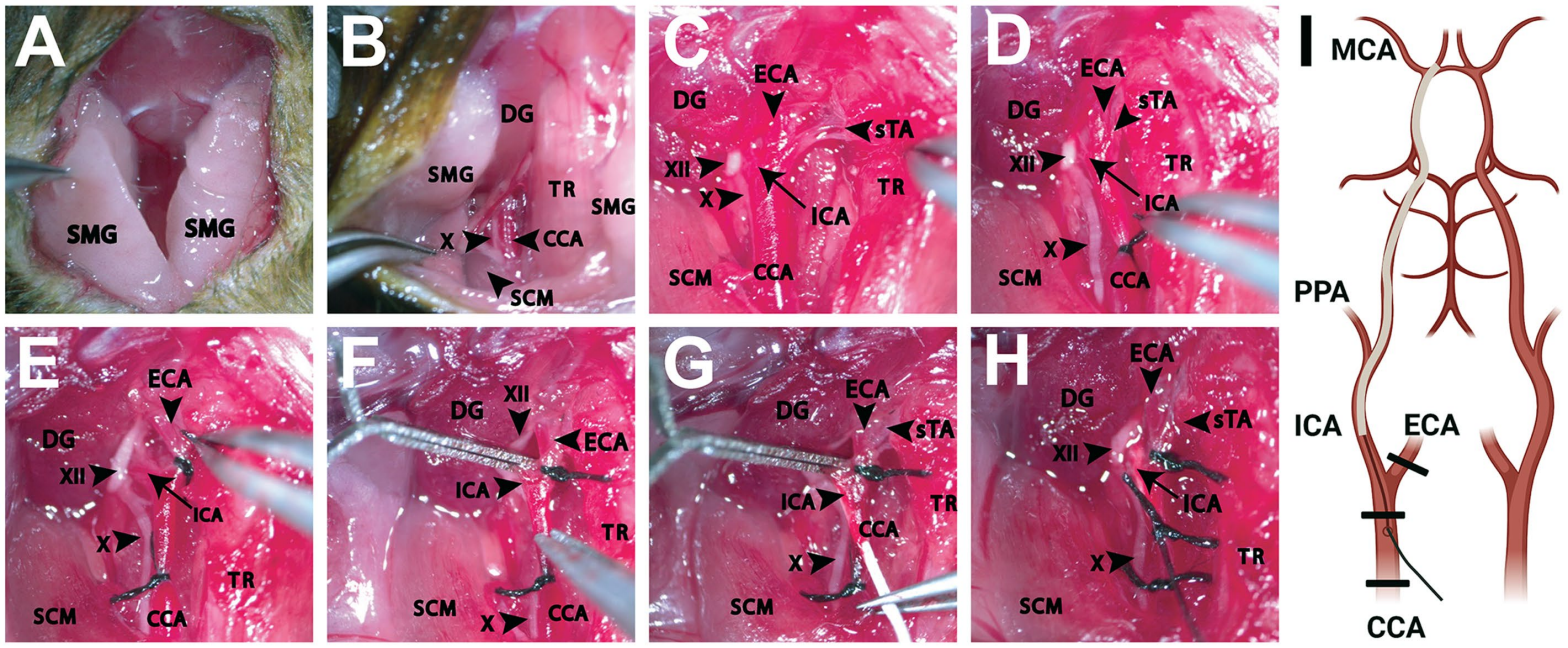
Dissection of the ventral neck region for intraluminal filament occlusion of the proximal middle cerebral artery. **(A)** Initial cervical incision made exposing the SMGs bilaterally. **(B)** Following retraction of the PGs and further blunt dissection, exposure of underlying anatomical structures is achieved. Adequate visualization of adjacent muscular landmarks including the SCM and DG aid in locating the CCA, which is often situated inferiorly to these structures. **(C)** Enhanced magnification and careful dissection reveal the CCA and its branches (ICA, ECA, sTA) as well as adjacent cranial nerves (X, XII). The TR can be retracted further medially as needed to better establish the surgical plane. **(D)** The CCA is then ligated (6–0, silk) with care being taken to avoid capturing the vagus nerve running inferolateral to the CCA. **(E)** A second ligation (6–0, silk) is performed around the ECA, inferior to the branch point of the sTA. **(F)** A temporary vessel clamp is then placed on the ICA, being careful to avoid the hypoglossal nerve running adjacent to the artery. Next, an arteriotomy is performed and any bleeding is addressed appropriately. **(G)** Once hemodynamic control is ensured, a silicone monofilament is then placed into the arteriotomy site and gently advanced toward the ICA branchpoint. **(H)** At this time, the temporary vessel clamp is removed, and the monofilament is advanced further into the ICA roughly 10 mm until the ICA-MCA branchpoint is reached. Care should be taken to not force the monofilament once met with resistance. An additional suture (6–0, silk) is utilized to secure and prevent retropulsion of the monofilament. **(I)** A schematic shows the relevant anatomy from a ventral view with the filament inserted into the right ICA to occlude the origin of the MCA vessel. Thicker black lines represent the location of the suture ties (Created with BioRender.com). CCA, common carotid artery; DG, digastric muscle; ECA, external carotid artery; ICA, internal carotid artery; MCA, middle cerebral artery; PPA, pterygopalatine artery; SCM, sternocleidomastoid muscle; SMG, submandibular gland; sTA, superior thyroid artery; TR, trachea; X, vagus nerve; XII, hypoglossal nerve.

The classic proximal MCAo model was initially developed by Koizumi et al. in rats, and it features the use of an intraluminal filament to obstruct blood flow to induce focal brain ischemia (Figure 1) (6). This model mimics a *large vessel occlusion* stroke as it occurs in humans, and the duration of ischemia is easily controllable allowing for adequate control of stroke parameters. Of note, flow through the ICA is not re-established in this model as it must be tied off for hemostasis, and reperfusion of the MCA territory occurs via collateral flow through the circle of Willis after removal of the filament. The Longa method involves slight variations to Koizumi’s method in order to reestablish flow through the ICA at the end of the procedure (7). In the Longa method, an ECA stump is used for filament insertion instead of the CCA, therefore on the removal of the filament, the ECA is tied off for hemostasis and flow through the CCA, and the ICA is re-established. Comparisons between these two models in mice show this leads to increased reperfusion after filament removal compared to the Koizumi technique. Interestingly, the Longa technique leads to a more robust inflammatory response than the Koizumi method as measured by leukocyte-endothelial interactions post-reperfusion (13). There is mixed data regarding whether there are significant differences in lesion volume or survival rates between the two techniques in mice (13, 14). A comparison done in rats showed the models led to similar infarct volume, mortality rate, and weight loss. However, the study did find the different models led to differences in interleukin-1B and corticosterone regulation (15) and a follow-up study showed only the Longa method led to a measurable increased memory deficit (16). There is a newer alternative approach that avoids ECA ligation or permanent occlusion through repair of the CCA arteriotomy at the end of the procedure through the use of fibrinogen and thrombin products, and this may decrease the infarct-size coefficient-of-variation (17). These models can easily be performed in either mice or rats (18, 19).

MCAo via these anterior cervical approaches to the CCA or ECA for filament insertion avoids some of the intracranial manipulations that may skew results, as opposed to other models that require craniotomies. Importantly, even if the ipsilateral cervical carotid circulation is permanently occluded, MCA reperfusion occurs after the removal of the filament. This is due to the redundant supply to the ipsilateral MCA through a robust circle of Willis. The proportion of the hemisphere that is infarcted is similar in mice and rats, and is dependent on the duration of ischemia, which is usually 30–90 min (18). Ultimately, these methods have the advantage that they allow for the study of transient ischemia and the effects of reperfusion.

### Distal middle cerebral artery occlusion

The first model describing the ligation of the MCA using an open craniectomy technique occurred in 1975 (20). Either permanent or transient MCA occlusion can be obtained. Commonly, permanent MCA occlusion is obtained via electrocauterization of the artery. Transient occlusion can be accomplished by the application of ligatures or microclips that can be removed to allow for reperfusion (8). The occlusion of the proximal MCA produces ischemic damage that is seen in the cortex of the frontal lobe and the lateral part of the caudate nucleus with some involvement of the sensory and auditory cortex. The more proximal the occlusion site of the MCA is, the larger and more consistent the infarct will be (9, 21). Using more distal MCA occlusions, there is less damage to the hypothalamus, hippocampus, and midbrain (22).

A successful occlusion of the MCA can be readily visualized with this technique, and the ability to induce transient or permanent ischemia provides flexibility. This technique does involve direct manipulation of and exposure of brain tissue, which makes the model less like the human disease. This manipulation can induce intracranial inflammation that could cause pathophysiologic responses distinct from stroke and could impact intracranial pressure and blood–brain barrier function. Nevertheless, confining ischemic injury to the neocortical areas is often considered a significant experimental advantage. Norat et al. used distal MCAo to measure stroke improvement after intraarterial transplantation of the mitochondria (23).

### Localized application of thrombin

As an alternative to mechanical ischemia, there are techniques that use pharmacologic means to induce ischemia and thrombogenesis that have been utilized in rodent models. The advantage of these models is the ability to study thromboembolic mechanisms and clot dynamics, and evaluate potential means of intervention to address these stroke mechanisms.

The thrombin model entails local injections of thrombin directly into the MCA of a mouse (10, 24). Thrombin, normally activated via the coagulation cascade in response to endothelial blood vessel injury, marks the initiation of secondary hemostasis. Thrombin catalyzes the polymerization of fibrinogen to fibrin, yielding a stable platelet-fibrin thrombus. Further structural stability is provided by factor XIIIa, which, activated by thrombin, promotes fibrin cross-linking and ultimately clot formation (25). By artificially inducing a thrombus, this model produces an occlusion that mimics a thromboembolic stroke. Applying the peptide endothelin-1 (ET-1) to blood vessels also causes strong and long-lasting vasoconstriction and hypoxia, which can block blood flow and lead to downstream ischemia. Furthermore, injecting ET-1 directly into brain tissue can induce prolonged focal ischemia (26). However, it should be noted that the particular type of injury caused by ET-1 is due to a distinct mechanism of constriction, rather than thrombosis or embolism, which are the main topics of this review.

The thrombin model procedure begins with a craniectomy and exposure of the right MCA for direct injection. If desired, IV-tPA can be administered through a tail vein catheter to induce thrombolysis and emulate vessel reperfusion (10, 24). A blood clot is considered successful if a 60% or more drop in CBF is observed via a laser Doppler flow probe. While a relatively simple procedure, there are some difficulties with this model. This includes spontaneous MCA recanalization, bleeding complications, and an inaccessible MCA bifurcation site. Additionally, upon further analysis of clot composition, it was found that clots contained primarily polymerized fibrin and a low number of cells and platelets. This is in contrast to humans in which clots consist of platelet/fibrin accumulation, linear neutrophil/monocyte deposition, and erythrocyte-rich accumulation (25).

There are drawbacks to these models, including the need to perform a craniotomy. In addition, there may be off-target effects. Otherwise, this is a useful model for assessing thrombolytic agents and their effectiveness in reducing infarction size in animals.

### Photothrombotic stroke model

The photothrombosis model involves using photo-oxidation to induce an infarct based on the interaction of an organic dye with light to facilitate platelet aggregation. The technique was first described in by Rosenblum and El-Sabban (27) and was later modified in 1985 by Watson et al. (11) Watson and colleagues introduced a photosensitive dye called Rose Bengal, which can be injected intraperitoneally or intravascularly. Upon illumination, the dye is activated and generates free radicals causing endothelial damage that leads to platelet activation and a thrombus formation (28). By using specific coordinates to guide irradiation, a researcher can determine a specific area in which to induce ischemia.

The benefit of this model is its ability to target accurate regions of interest using stereotactic coordinates. This results in the reproducibility of lesions and low mortality rates. Another unique advantage is that photothrombosis has been adapted to be applied in live rodents while they are awake and freely moving. In this model, the bone in the region of interest is thinned until the cortical vessels are visible and a cranial window is made and a head-mounted miniature stage is placed. Injury is induced using illumination through an optical fiber combined with an aspheric lens to target the region of interest. A lesion can be induced in 15 min. Combining a high spatiotemporal resolution imager with the head stage allows for real-time CBF imaging and analysis during the process of infarction (29).

Unfortunately, photothrombotic injury differs from a stroke in humans in that it involves a relatively large number of vessels in the illuminated area. This is in contrast to human stroke where ischemia often is a result of interrupted blood flow of a single terminal artery. Photocoagulation insult also causes severe blood vessel damage and substantial early vasogenic edema that is not generally characteristic of human stroke (28). However, there is a newer adaptation of this method that uses artery-targeted photothrombosis, which confines illumination to desired arterial branches and minimizes off-target damage to neighboring tissue (30). Finally, classically, photothrombosis-mediated clots are highly refractory to tPA-mediated lytic treatment, presumably due to the predominance of platelets and fibrin-poor clot composition. Recent work has been done to circumvent this by creating a model that combines rose bengal with thrombin to produce fibrin-enriched and tPA-sensitive clots (31).

## Optimizing stroke models for enhanced reproducibility

A widely held perspective on neuroprotective stroke research points out that treatments that are effective in animals often fail to show similar success in humans (32). In fact, hundreds of potential stroke treatments entered clinical trials based on promising preclinical data, but only recanalization therapies were successful (5). Notably, thrombolysis with tissue plasminogen activator (tPA), the only clinically effective pharmacological treatment of acute ischemic stroke, was first demonstrated and evaluated in an experimental model of stroke (33). The Stroke Preclinical Assessment Network (SPAN), a large research project funded by the National Institutes of Health, was developed to address the need for a better understanding of stroke research variability in rodent models (5).

In a significant advancement, a recent study from the 6 SPAN laboratories examined the heterogeneity caused by differences in biological and experimental model variables, as well as their impact on the MCAo performance (34). Factors such as age, time of day when MCAo was performed, choice of filament, maintaining anesthesia during occlusion, cerebral blood flow monitoring, and circadian stage of the animal at the time of MCAo were considered. Embracing this biological and methodological heterogeneity could better inform clinical trials, thereby enhancing the predictive value of preclinical testing. Furthermore, understanding the sources of heterogeneity and their effects on study performance may help refine study design and statistical modeling for future multicenter preclinical trials (17, 34, 35).

Candelario-Jalil and Paul have also discussed recent findings that emphasize the notable differences in stroke outcomes between young and aged animals, and how major comorbid conditions, such as hypertension, diabetes, obesity, and hyperlipidemia, significantly increase the brain’s vulnerability to ischemic damage, leading to worse functional outcomes. The review indicates that incorporating animal models of aging and comorbidities during the initial stages of drug development would aid in identifying neuroprotective strategies with a higher chance of success in stroke clinical trials (36). Also, there are big differences between rodent and larger animal stroke models, and using larger animals may resemble human strokes more closely. However, these models come with practical issues and ethical concerns that limit their use in research. The debate continues about whether using these models can help improve science and create treatments for human strokes (37).

Among the previously identified common factors, the failure to translate preclinical studies in rodents to clinical settings can be attributed to insufficient statistical power, poor experimental design, publication bias, lack of randomization and blinding in many preclinical studies, and an unrealistic therapeutic time window. Numerous recent articles and commentaries have addressed the primary causes of this translational roadblock in stroke research and proposed potential solutions to overcome it (38–42).

One crucial aspect of preclinical stroke research reproducibility involves monitoring ischemic injury and imaging the infarction. Advanced stroke imaging techniques, such as magnetic resonance imaging, serve as indispensable tools that allow researchers to visualize, map, and track the pathological changes in the brain post-stroke. These methods offer high-resolution images of brain structures and facilitate the identification and quantification of stroke lesions and related tissue damage, encompassing alterations in blood flow, edema, inflammation, and cellular injury. This data fosters a more precise comprehension of stroke processes, including the mechanisms behind stroke injury and repair. Consequently, the availability of advanced imaging methods significantly influences the reproducibility of targeted stroke treatments.

## Stroke imaging and infarct analysis in animal models

Preclinical stroke research heavily depends on imaging and quantification methods to accurately determine infarct and penumbra volumes. The imaging approaches include radiological methods such as computed tomography (CT), positron emission tomography (PET), and Magnetic Resonance imaging (MRI) (43). The optical techniques involve Laser Speckle Contrast Imaging (LSCI) technology, Photoacoustic Imaging (PAI), and 2-photon laser scanning microscopy (2PLSM) (44). The traditional histological methods are also enhanced by new advancements in genetic and immunohistochemical strategies.

### *In vivo* imaging of ischemic stroke and cerebrovascular disease

A variety of sensitive and specific radiology imaging techniques that are used for diagnostic purposes in patients are also available for imaging of the rodent brain. In addition to CT, MRI is particularly well suited for imaging stroke pathology and several optimized sequences have been established which can be used if the proper small animal scanners are available (43, 45).

**Figure 2:**
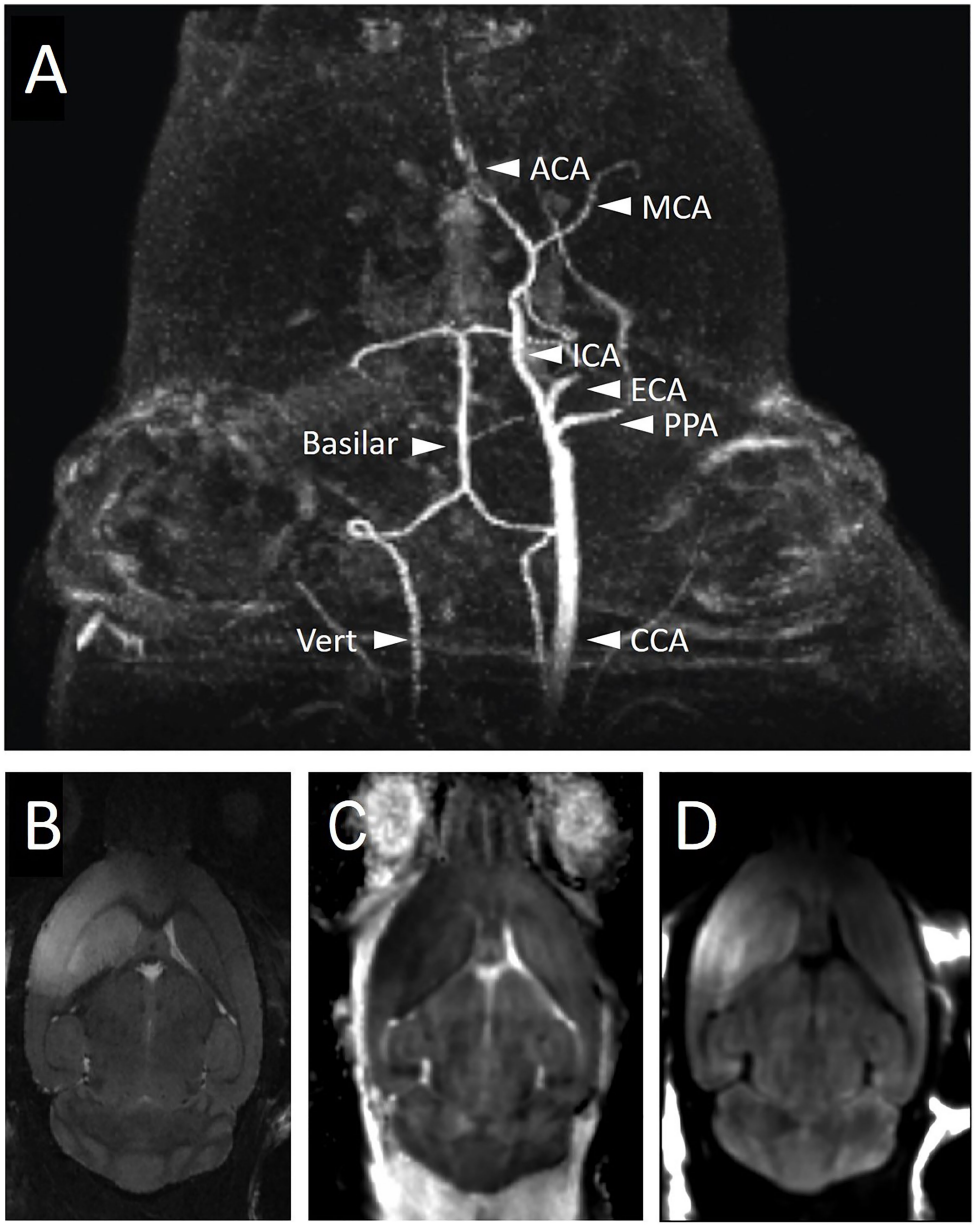
Imaging of mouse brain during and after right-sided MCAo with magnetic resonance imaging. **(A)** MRA during filament occlusion shows the absence of right-sided carotid circulation while the filament is in place. The intact posterior circulation and the left-sided carotid circulation are labeled. Mice were subjected to MCAo via filament for 1 h followed by filament removal and then 24 h of recovery time prior to the acquisition of T2 **(B)**, ADC **(C)**, and DWI **(D)** MRI sequences to visualize the infarct region. The infarct region in the right cortex and striatum is bright on T2 **(B)** and DWI **(D)** sequences and dark on ADC **(C)**. ACA, anterior cerebral artery; CCA, common carotid artery; ECA, external carotid artery; ICA, internal carotid artery; MCA, middle cerebral artery; PPA, pterygopalatine artery; Vert, vertebral artery.

CT is a quick, effective way to evaluate the presence of hemorrhage within the brain and can give an estimate of infarct territory in a subacute setting, but CT is inferior to MRI in its ability to identify infarct in the acute phase. MRI is the gold standard for assessing perfusion and ischemic injury in humans as it has the highest sensitivity and resolution. It is the modality that can most accurately predict regions at risk of ischemic injury and assess blood flow. Vessel imaging can be employed to confirm the location of large vessel occlusions, for example via magnetic resonance angiogram (MRA) (46), readily detecting MCA occlusion in the mouse brain (Figure 2A). On the other hand, diffusion-weighted imaging (DWI) and computed apparent diffusion coefficient (ADC) maps show regions of restricted diffusion, which estimates the core (Figures 2B–D) (47). MRI can be thus used to estimate the size of an infarct and can do so in the acute as well as subacute phase (48). Specifically, diffusion-weighted imaging (DWI) provides the ability to differentiate cytotoxic edema from vasogenic edema, something that CT and other MRI sequences including the T2-weighted MRI are unable to accomplish and limits their utility to distinguish ischemic tissue from edema (49). DWI also helps with establishing the age of an infarct as diffuse characteristics evolve with time from high signal to normal signal and finally to low signal (50). Diffusion imaging combined with perfusion imaging has the potential to identify ischemic changes to brain tissue that are reversible with the intervention (51).

Further, Perfusion-Weighted Imaging (PWI) MRI can identify areas with decreased perfusion in order to estimate tissue at risk of ischemia, the penumbra. The more sensitive perfusion technique to measure penumbra requires contrast and is known as the dynamic susceptibility contrast-enhanced (DSC) MR perfusion (52). This is a contrasted T1 sequence that measures relative cerebral blood volume, relative cerebral blood flow and mean transit time. It is more sensitive than Arterial Spin-Labelling (ASL) MR perfusion which is a technique that capitalizes on the ability of MRI to magnetically label arterial blood so that it essentially creates a tracer to measure cerebral blood flow (53). Work has been done to develop these protocols for use in rodents (54). Unlike CT imaging, for which acquisition can take seconds, MRI acquisition takes minutes per sequence, and therefore, animals must be completely immobilized to perform this imaging in order to obtain adequate resolution (55). This requires general anesthesia, and as mentioned above, this has the potential to alter biological processes and confound outcomes.

Optical imaging technologies are common in academic research but until recently only a few have been translated into the clinic. Among the wide-field imaging techniques, Laser Speckle Contrast Imaging (LSCI) is nevertheless gaining popularity as a versatile flow imaging technique based on the analysis of light speckle pattern fluctuations, which allows the analysis of tissue perfusion with blood (56). In mice, LSCI offers the ability to measure relative cerebral blood flow through the intact skull. LSCI can monitor the middle cerebral artery occlusion and reperfusion stroke model (57). LSCI technology compares favorably to Laser Doppler Flowmetry (LDF) which used to be a prevalent method to monitor real-time cortical perfusion in rodents. This technique measures the Doppler effect, analyzing wavelength shifts of the reflected light as it scatters off moving cells in blood vessels, thereby quantifying the rate of cerebral blood flow (58, 59). These measurements are evidently of paramount importance in ensuring the best possible reproducibility of research in ischemic stroke.

Wide-field fluorescence imaging also enables fundamental insights into functional recovery from ischemic injury. This technique, sometimes called Wide-field Functional Optical Imaging (WFOI), is employed in rodent models of stroke, primarily in the mouse. WFOI requires minimally invasive surgery to expose the skull prior to imaging, and a small Plexiglas window attached to the intact skull for chronic imaging (60). Following a stroke, functional MRI studies in humans have shown that local brain circuits lost to infarction remap to the peri-infarct cortex and are more spatially focused in patients exhibiting more complete recovery (61). In rodents, remodeling of local circuitry in the periinfarct cortex correlates temporally with the behavioral recovery (62). Thus, information learned from functional WFOI neuroimaging can be used to inform interventional strategies designed to affect plasticity mechanisms after injury (63).

Photoacoustic Imaging (PAI) is a new imaging technique that monitors the anatomical, molecular, and metabolic features of biological tissues by identifying their optical absorption properties, using sound as a readout. The device sends non-harmful laser pulses into tissues, where some of the energy is absorbed and changed into heat, causing temporary expansion and ultrasound emission. Ultrasound waves are picked up by sensors and used to create images. PAI is very flexible and can examine the same process at different scales from single cells to entire organs. With hemoglobin as a natural marker, PAI can image blood vessels in the brain without any added labels and track blood-related properties such as oxygen levels and flow. This makes PAI very helpful when investigating changes in blood flow and blood oxygen supply caused by the stroke (64). The potential of this technique was demonstrated by tracking the vascular and metabolic responses in an awake mouse brain during acute, subacute, and chronic stages of ischemic stroke. A side-by-side comparison of the injured (ipsilateral) and control (contralateral) cortices revealed that, despite the early recovery of cerebral blood flow and increased microvessel density, a persistent deficit in cerebral oxygen metabolism was observed throughout the chronic stage in the injured cortex, leading to infarction. This advanced functional-metabolic imaging technique presents new possibilities for studying the long-term progression and treatment outcomes of neurovascular diseases (65).

Microscopic imaging using two-photon laser scanning microscopy (2PLSM) provides superior spatial and temporal resolution of specific pathological events associated with stroke-induced damage (44, 66). This technology utilizes pulsed, tunable infrared lasers, focused to about 1 cubic micron volume using high numerical aperture objectives and fast scanners (67). The resulting high photon density facilitates near-simultaneous absorption of two infrared photons in the focal volume, allowing the excitation and detection of visible dyes including green and red fluorescent proteins deep in the tissue (68). These approaches usually employ either a thinned skull or a glass-covered cranial window to perform *in vivo* imaging up to 500 microns deep. In combination with genetically encoded indicators of calcium or glutamate (69–71), 2PLSM has revealed a wealth of information about ionic shifts that are triggered during the cortical spreading depolarizations commonly seen in ischemic strokes (72, 73). Specifically, calcium transients in the neurovascular unit comprising neurons and astrocytes (74, 75), and more recently also microglia (76), have been characterized following stroke. Calcium overload plays a critical role in the pathophysiology of ischemic injury, leading to a cascade of detrimental effects including cell death. Further investigation of these processes is therefore highly warranted.

### Histological detection and evaluation of ischemic damage in the infarcted area

**Figure 3:**
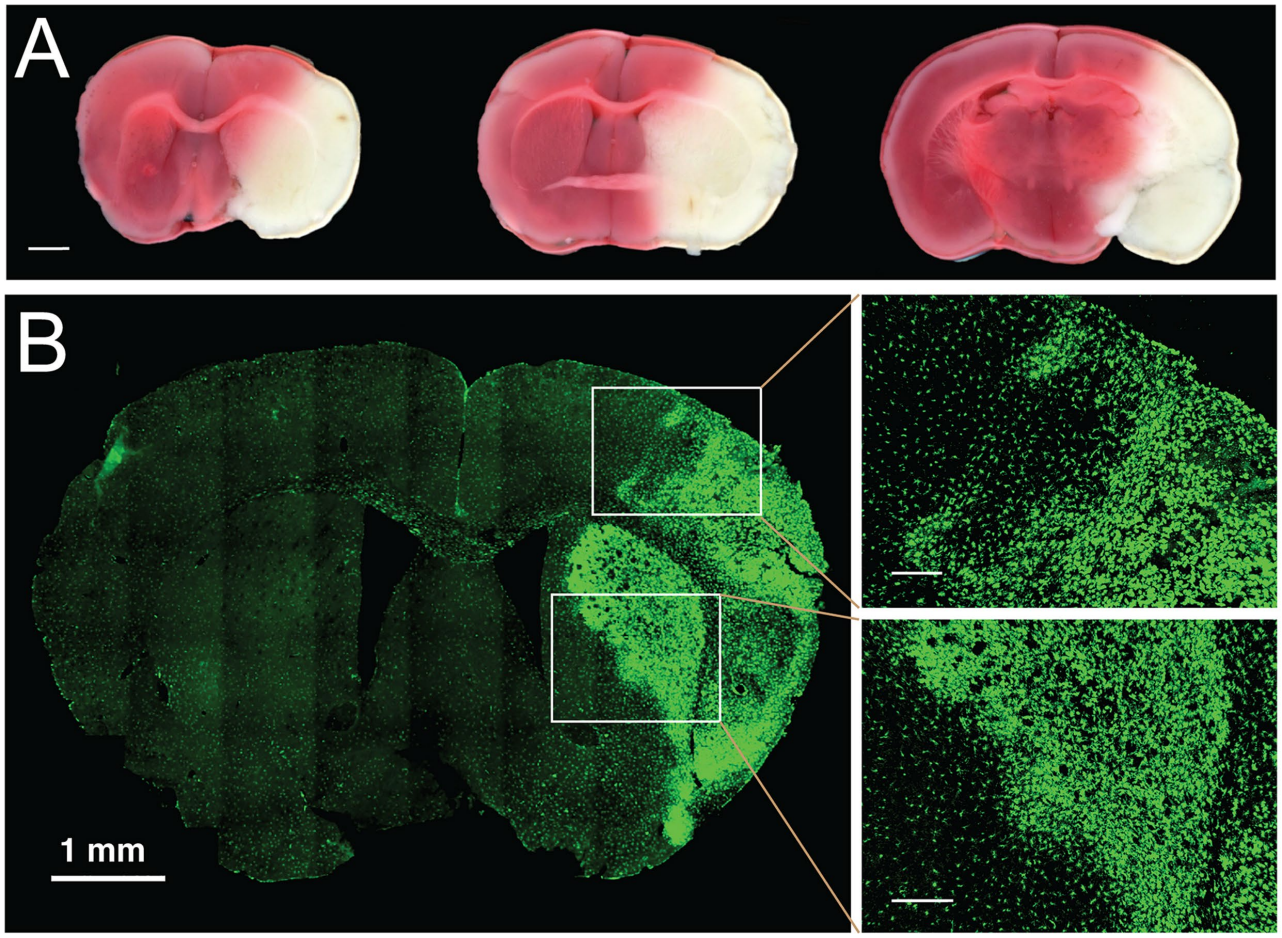
Histological and genetic cell lineage labeling of ischemic stroke injury. **(A)** Histological staining of live brain slices with TTC. Staining of freshly cut, 1-mm thick coronal brain slices with 2,3,5-Triphenyltetrazolium chloride (TTC) is commonly used to determine the size of the infarcted area in the brain. Three coronal planes are displayed from a brain with a permanently occluded middle cerebral artery and stained 24 h after stroke induction. White areas delineate the infarcted tissue, red color indicates the tissue with normal mitochondrial activity. Scale bar, 1 mm. **(B)** Genetic labeling of chronic infarction following focal ischemic stroke. Transient MCAo was performed in a transgenic mouse model expressing the Aif1-Dre allele and the RC::RLTG Dre/rox reporter (78). In this reporter system, Iba1-positive myeloid cells are robustly stained with antibodies against the lineage marker (tdTomato), shown in green. One week after transient occlusion, the labeling identifies the infarcted area by the morphological transformation of microglia and border macrophages. The panels on the right-side show in detail the morphological transition of myeloid cells at the border of chronic infarction in the cortex (right top) and in the striatum (right bottom). Scale bars in enlarged panels, 200 μm.

The size of an infarct has been traditionally estimated using histological techniques. There are many different staining techniques used to assess the extent of ischemic injury, each with benefits and drawbacks. Common staining methods that are frequently used in stroke experiments include 2,3,5-triphenyltetrazolium chloride (TTC) and Fluoro-Jade B, in addition to the other classic stains such as Nissl, Hematoxylin and Eosin (H&E) and Cresyl Violet (CV) (77). TTC staining continues to be a mainstay for visualizing stroke injury in rodent models (Figure 3A). However, drawbacks of TTC include that it must be done immediately on fresh tissue and it may not truly label irreversible cell death as it is a marker of mitochondrial dysfunction and tissue dehydrogenase activity; thus, it may overestimate cell death (79, 80). An alternate method for staining is Fluoro-Jade B. This stain is an anionic dye that stains the soma and neurites of degenerating neurons, and it has been validated as a useful indicator in the acute cerebral ischemia (77). A drawback of Fluoro-Jade B is that the mechanism of labeling has not been elucidated, and it is unclear what underlying physiological process is indicated by staining. A disadvantage of both of these markers is that there is limited ability to define core versus penumbra and to perform colocalization experiments. There is a need for markers that more explicitly define the injury as apoptotic versus necrotic and allow co-labeling, for example, to characterize the immune response within and around the infarct.

Further insights into the pathology of ischemic brain injury and recovery are gained through cell fate mapping experiments with genetic labeling tools such as Cre, Dre, FLP, or other site-specific recombinases (78). Genetic labeling of immune cells can be particularly illuminating because these cell populations undergo a rapid and profound transformation in the infarcted area. For example, genetic labeling with the *Aif1-Dre* allele (similar to *Aif1-Cre* (81)) and the Dre/rox reporter line (78) robustly delineates the activation of the Iba1-positive myeloid cell population in the infarcted area after MCAo (Figure 3B). Further improvements in the resolution of cell fate mapping of ischemic injury will be achieved by dual recombinase-mediated approaches, whereby two gene promoters, and consequently two recombinases (e.g., Cre and Dre) identify specific cell types through intersectional or subtractive fate mapping with appropriately designed genetic reporters (82).

**Figure 4:**
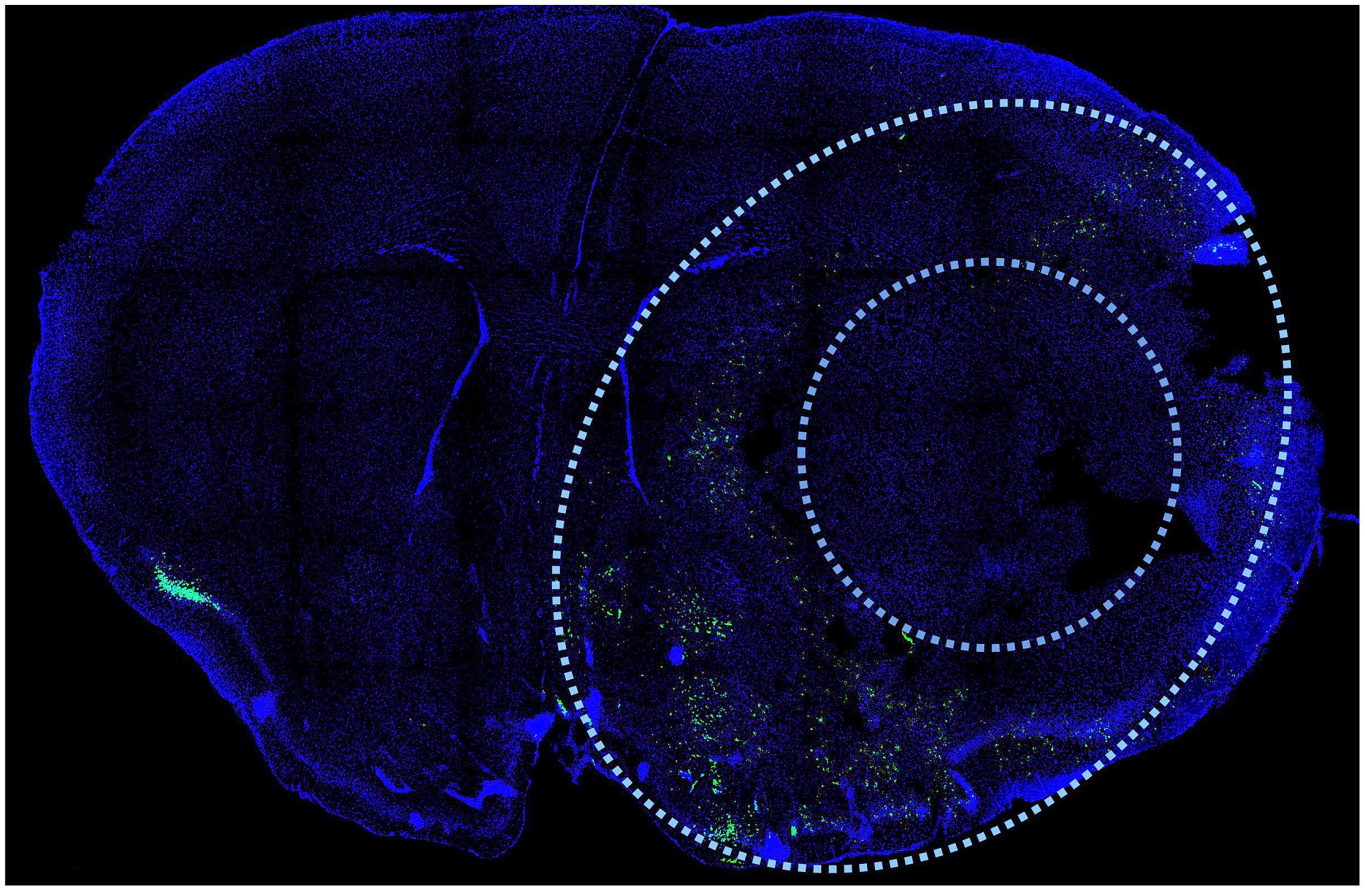
Immunodetection of cell death after MCAo. Antibodies against activated caspases and caspase substrates provide information about the nature of the injury as they indicate activation of the apoptotic cascade and can be used to indicate apoptotic cells in the penumbra region after stroke. One such antibody is specific to a caspase-cleaved fragment of actin, known as fractin. This antibody marker is highly sensitive and specific to apoptotic cells and is able to label cell bodies as well as axons and dendrite of dying neurons after stroke (83). Fractin (green) distinguishes the penumbra (outer limit defined by outer oval) from the necrotic core (inner circle) as it highlights apoptotic cells in the region of the penumbra 24 h after transient MCAo. Coronal section prepared as described (83). DAPI (blue) is a stain that highlights nuclei.

Immunohistochemistry is another versatile and informative method for characterizing infarct injury. Antibodies against activated caspases and caspase substrates provide some information about the nature of the injury as they indicate activation of the apoptotic cascade and can be used to indicate apoptotic cells. One such antibody is specific to a caspase-cleaved *fragment* of *actin*, known as *fractin* (83). This antibody marker is highly sensitive and specific to apoptotic cells and is able to label cell bodies as well as axons and dendrite of dying neurons. *Fractin* highlights apoptotic cells in the region of the penumbra 24 h after transient MCAo and can be used to estimate the size of the core and penumbra (Figure 4). Antibodies to caspase substrates, like *fractin*, can be used alongside other immunolabeling markers, for example, to examine the localization of immune cells around the infarct. A drawback of antibody labeling is that epitopes may be transient and once cell death has proceeded to necrosis, the epitopes will be degraded.

## Conclusion

Rodent models of ischemic stroke appeal to researchers due to their ease of use and manipulability along with the ability to replicate experiments at economically reasonable costs. Middle cerebral artery occlusion remains the gold-standard approach for modeling cerebral vessel occlusion and recanalization. Selecting the right model for the experiment is a crucial step; when choosing a particular stroke model, factors to consider include desired infarct size and ability to reperfuse, as well as resultant penumbra: core ratios, behavioral deficits, inflammatory responses, and mortality rates.

There are significant limitations associated with the use of rodents for the study of stroke, including their distinct cerebral architecture, and variations in their thrombotic, inflammatory and cell death cascades compared to humans. However, advances in imaging in conjunction with immunohistochemical staining and the use of transgenic mouse models facilitates more pointed investigation, characterization, and interpretation of stroke processes. Regardless, interventions with promising results in rodents must be subject to further validation before assuming they will translate to success in human studies.

## Author contributions

JS, SS, and PN: acquisition, analysis, or interpretation of data for the work, drafting the work or revising it critically for importance. KS, KK, LL, AW, and GM: acquisition and analysis. KY and PM: analysis and interpretation of data for the work. MK, MP, and RK: interpretation of data for the work. PT: analysis, interpretation of data, drafting the work or revising it critically for importance. All authors contributed to the article and approved the submitted version.

## Funding

This work was supported by NIH R21NS116431 and grants from the Focused Ultrasound Foundation (FUSF) to P. Tvrdik. J. D. Sokolowski was supported with funding from the Neurosurgery Research and Education Foundation (NREF).

## Conflict of interest

The authors declare that the research was conducted in the absence of any commercial or financial relationships that could be construed as a potential conflict of interest.

## Publisher’s note

All claims expressed in this article are solely those of the authors and do not necessarily represent those of their affiliated organizations, or those of the publisher, the editors and the reviewers. Any product that may be evaluated in this article, or claim that may be made by its manufacturer, is not guaranteed or endorsed by the publisher.
